# Age-Related Associations of Blood Pressure and Arterial Stiffness with Retinal Arterial Structure

**DOI:** 10.1016/j.xops.2025.100957

**Published:** 2025-10-06

**Authors:** Ran Xiang, Yuki Muraoka, Takahiro Kogo, Yu Hidaka, Yuki Mori, Masayuki Hata, Masahiro Miyake, Satoshi Morita, Yasuharu Tabara, Fumihiko Matsuda, Akitaka Tsujikawa

**Affiliations:** 1 Department of Ophthalmology and Visual Sciences, Graduate School of Medicine, Kyoto University, Kyoto, Japan; 2 Department of Biomedical Statistics and Bioinformatics, Graduate School of Medicine, Kyoto University, Kyoto, Japan; 3 Institute for Advancement of Clinical and Translational Science, Kyoto University Hospital; 4 Graduate School of Public Health, Shizuoka Graduate University of Public Health, Shizuoka, Japan; 5 Center for Genomic Medicine, Kyoto University Graduate School of Medicine, Kyoto, Japan

**Keywords:** OCT, Retinal arterial structure, Systolic blood pressure, Arterial stiffness, Nagahama study

## Abstract

**Objective:**

To investigate the associations of systolic blood pressure (SBP) and arterial stiffness, measured via the cardio-ankle vascular index (CAVI), with retinal arterial structure assessed by OCT, and examine age-related differences.

**Design:**

A cross-sectional analysis of a community-based Japanese cohort.

**Participants:**

A total of 6969 adults (mean age, 57.6 years; 69.6% women) who underwent OCT imaging between 2012 and 2016.

**Methods:**

Peripapillary circular OCT B-scans were used to assess the 4 largest retinal arteries. OCT-derived parameters included outer diameter (OD), inner diameter (ID), wall thickness, and wall reflectivity. Multivariable linear regression adjusted for demographic, ocular, and systemic covariates. Interaction analyses evaluated age modification, followed by analyses stratified by age (<65 vs. ≥65 years).

**Main Outcome Measures:**

OCT-based measurements of OD, ID, wall thickness, and wall reflectivity.

**Results:**

Higher SBP was associated with smaller OD (β = –0.136, 95% confidence interval [CI]: –0.151 to –0.120) and ID (β = –0.136, 95% CI: –0.150 to –0.121). The CAVI was positively associated with wall reflectivity (β = 0.371, 95% CI: –0.081 to 0.822). Interaction analyses indicated age-related modification (SBP × age for OD: β = 0.005; ID: β = 0.005; CAVI × age for wall reflectivity: β = 0.030). In stratified analyses, the inverse associations of SBP with OD and ID were stronger in participants aged <65 years (OD: β = –0.186; ID: β = –0.178) than in those aged ≥65 years (OD: β = –0.076; ID: β = –0.085). For CAVI, no clear association with wall reflectivity was found in participants aged <65 years (β = –0.165), whereas a positive association was observed in those aged ≥65 years (β = 0.725).

**Conclusions:**

OCT-based retinal arterial measurements revealed age-dependent associations with systemic vascular factors. In younger adults, elevated SBP was linked to narrower arterial diameters, reflecting functional vasoconstriction, whereas in older adults, greater wall reflectivity was associated with arterial stiffness, suggesting structural remodeling. These findings support OCT as a noninvasive tool for assessing different stages of microvascular aging, warranting confirmation in longitudinal studies.

**Financial Disclosure(s):**

Proprietary or commercial disclosure may be found in the Footnotes and Disclosures at the end of this article.

Arterial health reflects both functional and structural changes in the vasculature. Age-related alterations such as medial thickening, fibrosis, and calcification contribute to progressive arterial stiffening and are generally considered irreversible.^1^^,^^2^ In contrast, early-stage functional abnormalities, including increased vascular tone and endothelial dysfunction, are potentially reversible and may improve with appropriate interventions.^3^ Although vascular aging has been well characterized in large arteries, mounting evidence indicates that the microvasculature also undergoes parallel functional and structural remodeling.^4^^,^^5^ These microvascular changes have gained increasing recognition as readily measurable indicators of systemic vascular health.

The retina provides a uniquely accessible and noninvasive window into the microcirculation.^6^ Given its structural and physiological similarity to the cerebral and renal microvasculature, retinal vessel morphology is considered a surrogate marker of systemic vascular status.^7^ Changes in retinal vessel caliber are reportedly associated with hypertension, stroke, and cardiovascular disease.^8^, ^9^, ^10^ However, traditional fundus photography can only offer two-dimensional images, limiting evaluation of the vessel wall.^11^^,^^12^

OCT enables high-resolution, cross-sectional imaging of the retina, allowing quantitative analysis of vascular features, including the outer diameter (OD), inner diameter (ID), wall thickness, and wall reflectivity.^13^^,^^14^ These parameters may reflect microvascular remodeling—an early manifestation of vascular injury associated with hemodynamic stress. Population-based studies have linked OCT-derived vessel metrics with demographic and systemic factors, highlighting their potential relevance in evaluating vascular health.^14^

Among these OCT-derived parameters, wall reflectivity represents signal intensity within the vessel wall and may reflect early structural changes in the microvasculature.^14^^,^^15^ However, its physiological and pathological relevance remains to be clarified. Arterial stiffness, as assessed by the cardio-ankle vascular index (CAVI), is a validated noninvasive measure linked to cardiovascular outcomes in diverse populations.^16^^,^^17^ However, its relationship with retinal vascular structure has not been systematically investigated.

In this large-scale, community-based cohort study, we investigated the associations of systolic blood pressure (SBP) and arterial stiffness, measured via the CAVI, with OCT-derived retinal arterial parameters. We further examined whether these associations varied according to age. In particular, we investigated whether vessel caliber narrowing and increased wall reflectivity could reflect functional and structural vascular responses, respectively.

## Methods

### Study Population and Data Collection

This cross-sectional study utilized data from the Nagahama Prospective Cohort for Comprehensive Human Bioscience, a large, community-based cohort of approximately 10 000 residents of Nagahama City, Japan. The cohort was designed to investigate environmental and genetic factors influencing health and has been previously used for research in the systemic and ophthalmic fields.^14^^,^^18^^,^^19^ For the current analysis, data from the second survey cycle (2012–2016) were used. This included comprehensive health assessments and ophthalmic imaging performed approximately 5 years after baseline. This study was approved by the Ethics Committees of Kyoto University Graduate School of Medicine and Nagahama Municipal Hospital. The research adhered to the tenets of the Declaration of Helsinki, and written informed consent was obtained from all participants.

The exclusion criteria included a history of ocular surgery other than cataract surgery; high myopia (axial length >26 mm); hyperopia (axial length <21 mm); major retinal diseases (e.g., epiretinal membrane, macular hole, diabetic retinopathy, retinal vein occlusion, age-related macular degeneration, or retinoschisis); glaucoma or other optic nerve diseases; pregnancy; poor-quality OCT images; or missing relevant ocular or systemic data.

### Systemic and Ophthalmic Assessments

Systemic and ophthalmic parameters were assessed using standardized protocols. Blood pressure was measured after 5 minutes of seated rest twice using an automated oscillometric device; the mean of the 2 readings was used. Arterial stiffness was evaluated by measuring the CAVI using a validated device (Vasera-1500; Fukuda Denshi Co, Ltd) according to manufacturer-recommended protocols.^20^ Based on thresholds established in a previous study,^21^ CAVI was categorized as normal (<8.0), borderline (8.0–8.9), or abnormal (≥9.0).

Hypertension was categorized as normotensive (SBP <120 mmHg), elevated (SBP 120–129 mmHg), or hypertensive (SBP ≥130 mmHg) according to the American Heart Association classification.^22^ Information on alcohol consumption, history of smoking, and histories of hypertension, diabetes mellitus, dyslipidemia, and antihypertensive medication use was obtained from self-administered questionnaires. Alcohol consumption was recorded in grams per day. The respective histories of smoking, hypertension, diabetes mellitus, dyslipidemia, and antihypertensive medication use were classified as present or absent. The regular use of antihypertensive drugs was defined as antihypertensive medication use.

Ocular measurements included intraocular pressure (TX-20P; Canon), axial length (IOL Master; Carl Zeiss Meditec, Inc), and spectral-domain OCT (RS-3000 Advance; NIDEK).

### OCT-Based Vessel Assessments

**Figure 1:**
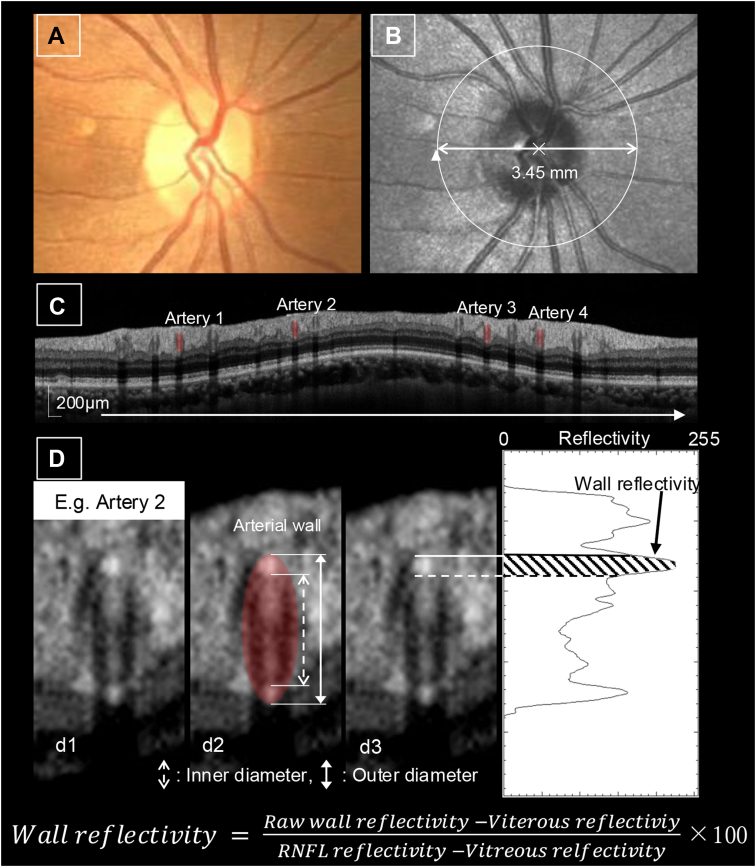
Quantification of retinal arterial parameters using OCT. **A,** Color fundus photograph. **B,** Scanning laser ophthalmoscopy image. The white circular arrow represents the location and direction of the circular OCT scan around the optic disc, which was used to acquire cross-sectional images of the retinal vessels. **C,** Circular OCT image around the optic disc. A representative OCT B-scan image shows cross-sectional views of the retinal vessels. Red ovals indicate arteries. **D,** Magnified images of retinal arterial cross-section. (d1) Original magnified image. (d2) Image showing the arterial outline and vessel wall boundaries. The OD and ID are measured from the delineated vessel boundaries. The wall thickness is calculated as half of the difference between the OD and ID. (d3) Measurement of arterial wall reflectivity with the plot profile. Arterial wall reflectivity is assessed within the inner vessel wall to minimize shadowing artifacts from the intraluminal blood content. Reflectivity values are normalized by using the signal intensities of the vitreous and retinal nerve fiber layers. ID = inner diameter; OD = outer diameter.

For each eye, the four largest retinal arteries were identified based on their OD, with reference to coregistered scanning laser ophthalmoscopy and color fundus photographs. Retinal vessel parameters were assessed using peripapillary circular B-scans (3.45 mm in diameter) centered on the optic disc (Fig 1), in accordance with the standardized protocol validated in previous studies using the Nagahama cohort.^14^^,^^23^

As reported by previous research,^14^ 4 arterial parameters were quantified from the OCT images: OD, ID, wall thickness, and wall reflectivity. The OD and ID were initially identified by a semiautomated algorithm based on vertical reflectivity profiles extracted perpendicular to the vessel axis, followed by manual refinement by trained examiners with reference to the reflectivity pattern on B-scan images. Wall thickness was calculated as half the difference between the OD and ID. Wall reflectivity was quantified along the inner vessel wall to minimize signal attenuation from intraluminal blood.

To account for interindividual variations in OCT signal intensity, wall reflectivity values were normalized using the vitreous body and retinal nerve fiber layer as internal references,^24^^,^^25^ as originally proposed by Borrelli et al in the context of age-related macular degeneration,^26^ using the following formula:

$$\text{Wall}\;\text{reflectivity}=\frac{\left(\text{Raw}\;\text{wall}\;\text{reflectivity}–\text{Vitreous}\;\text{body}\;\text{reflectivity}\right)}{\left(\text{RNFL}\;\text{reflectivity}–\text{Vitreous}\;\text{body}\;\text{reflectivity}\right)}\times 100$$

For each artery, reflectivity was measured along a central perpendicular line and two adjacent lines 300 μm apart; the average value from the central line was designated as the raw wall reflectivity. The final value was calculated as the average of values for the four largest arteries in each eye.

Previous studies have demonstrated high reproducibility of this image analysis protocol, with intraclass correlation coefficients ranging from 0.767 to 0.924 for intraobserver agreement and from 0.815 to 0.957 for interobserver agreement.^14^

### Statistical Analysis

Descriptive statistics were presented as frequencies and proportions for categorical variables and means and 95% confidence intervals (CIs) for continuous variables.

The association between retinal arterial parameters and systemic vascular indices (SBP and CAVI) was analyzed using multivariable linear regression models, with OD, ID, wall thickness, and wall reflectivity as dependent variables. The covariates included age, sex, body mass index, axial length, intraocular pressure, and self-reported lifestyle and clinical factors (alcohol consumption, smoking history, and histories of hypertension, diabetes, dyslipidemia, and antihypertensive medication use), which are associated with retinal vascular parameters, as shown by previous studies.^14^^,^^27^^,^^28^

Because of high collinearity between SBP and diastolic blood pressure (DBP) (Pearson *r* = 0.75; variance inflation factor for DBP = 3.1), SBP and DBP were modeled separately.

Based on the observed age-related associations of SBP and CAVI, formal interaction testing was conducted to assess effect modification by age. The interaction terms, SBP × age and CAVI × age, were included in the models to evaluate whether these relationships varied by age.

To better characterize potential age-related differences in vascular associations, stratified analyses were performed by age (<65 vs. ≥65 years). A threshold of 65 years was chosen based on the World Health Organization's definition of older adults and previous studies on vascular health, which have reported differences in microvascular structure and function around this age.^29^

For all primary and stratified analyses, the estimated β coefficients and 95% CIs were reported; *P* values were not presented, and interpretation was based on the magnitude and direction of the effect estimates. All analyses were conducted using JMP Pro, version 18.0.2 (JMP Statistical Discovery LLC).

## Results

### Participants' Baseline Characteristics

**Figure 2:**
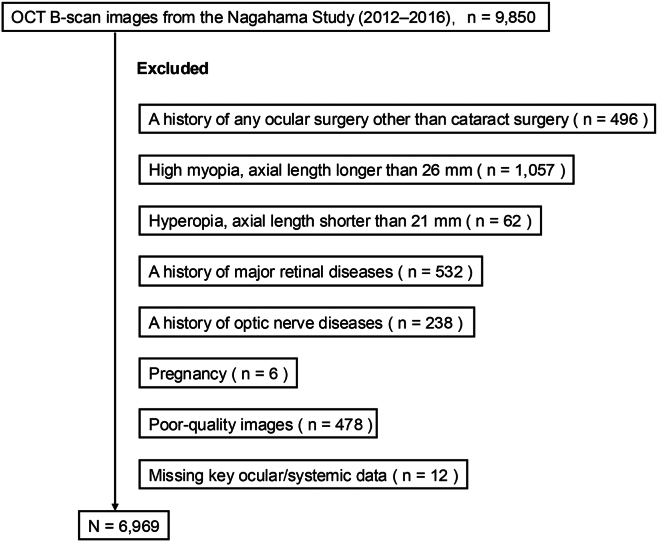
Flow diagram of participants. Flow diagram showing the number of participants included and excluded in the study. The reasons for exclusion are listed. The final analysis was performed with 6969 participants.

Among the 9850 participants (age 34–80 years) who underwent ophthalmic examinations, we excluded those with a history of ocular surgery other than cataract surgery (n = 496), high myopia (axial length >26 mm; n = 1057), hyperopia (axial length <21 mm; n = 62), a history of major retinal diseases (n = 532), a history of optic nerve diseases (n = 238), pregnancy (n = 6), poor-quality OCT images (n = 478), or missing key ocular/systemic data (n = 12), resulting in a final sample of 6969 participants (Fig 2). Table 1 summarizes the baseline characteristics. The participants' mean age was 57.6 years, and 69.6% were women. Overall, 66.7% of participants were aged <65 years, and 33.3% were aged ≥65 years.

**Table 1:** Participants' Characteristics (n = 6969)

Sex, n (%)	
Male	2121 (30.4%)
Female	4848 (69.6%)
Age, yrs	57.6 (57.3–57.9)
<65 yrs, n (%)	4648 (66.7%)
≥65 yrs, n (%)	2321 (33.3%)
Body mass index, kg/m^2^	22.19 (22.11–22.27)
Axial length, mm	23.78 (23.76–23.81)
Intraocular pressure, mmHg	14.2 (14.2–14.3)
Systolic blood pressure, mmHg	123.7 (123.2–124.1)
Normotensive (<120.0 mmHg), n (%)	3102 (44.5%)
Elevated (120.0–129.9 mmHg), n (%)	1494 (21.4%)
Hypertensive (≥130.0 mmHg), n (%)	2373 (34.1%)
Cardio-ankle vascular index	7.73 (7.70–7.76)
Normal (<8.0), n (%)	4323 (62.1%)
Borderline (8.0–8.9), n (%)	1565 (22.5%)
Abnormal (≥9.0), n (%)	1078 (15.5%)
Alcohol consumption, g/day	19.3 (18.6–20.0)
Smoking history (Yes/No)	2214 (31.8%)/4755 (68.2%)
Hypertension history (Yes/No)	1801 (25.8%)/5168 (74.2%)
Diabetes mellitus history (Yes/No)	440 (6.3%)/6529 (93.7%)
Dyslipidemia history (Yes/No)	1492 (21.4%)/5477 (78.6%)
Antihypertensive medication use (Yes/No)	1525 (21.9%)/5444 (78.1%)

Values are presented as the mean (95% confidence interval), unless indicated otherwise.

Systolic blood pressure was classified according to the American Heart Association guidelines.

The cardio-ankle vascular index was classified based on previous studies and cohort distribution.

The mean SBP was 123.7 mmHg. In this cohort, 44.5% of participants were normotensive, 21.4% had elevated blood pressure, and 34.1% were hypertensive.

The mean CAVI was 7.73. In this cohort, 62.1% of participants were classified as having normal CAVI, 22.5% were classified as borderline, and 15.5% were classified as having abnormal arterial stiffness.

### Associations of OCT-Based Retinal Arterial Parameters with SBP and CAVI

**Figure 3:**
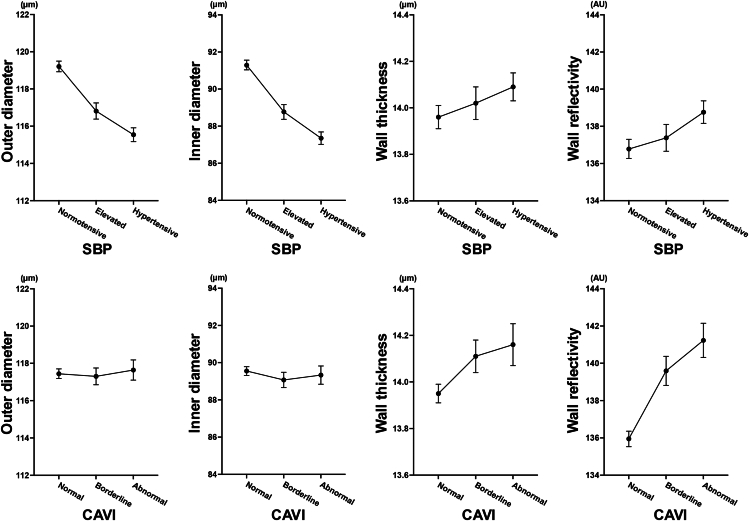
Associations of retinal arterial parameters with SBP and CAVI in the entire cohort. The four OCT-derived retinal arterial parameters—OD, ID, wall thickness, and wall reflectivity—across categories of SBP and CAVI are presented. Participants with higher SBP levels tend to exhibit smaller ODs and IDs, whereas those with higher CAVI values show a visual trend toward increased wall reflectivity. Systolic blood pressure was categorized according to the American Heart Association guidelines: <120 mmHg (normotensive), 120–129 mmHg (elevated), and ≥130 mmHg (hypertensive). The classification of CAVI was based on previous studies and the distribution within this cohort: <8.0 (normal), 8.0–8.9 (borderline), and ≥9.0 (abnormal). Error bars represent mean values with 95% confidence intervals. CAVI = cardio-ankle vascular index; ID = inner diameter; OD = outer diameter; SBP = systolic blood pressure.

The mean values and 95% CIs of OD, ID, and wall reflectivity across categories of SBP and CAVI are shown (Fig 3). Participants with higher SBP tended to have smaller OD and ID, whereas those with higher CAVI tended to have greater wall reflectivity.

Multivariable linear regression (Table 2) revealed that SBP was associated with smaller OD (β = –0.136, 95% CI: –0.151 to –0.120) and smaller ID (β = –0.136, 95% CI: –0.150 to –0.121). The CAVI showed little association with OD (β = –0.150, 95% CI: –0.408 to 0.109), ID (β = –0.135, 95% CI: –0.373 to 0.102), or wall thickness (β = –0.007, 95% CI: –0.050 to 0.036) but a relatively large positive association with wall reflectivity (β = 0.371, 95% CI: –0.081 to 0.822).

**Table 2:** Associations of Retinal Arterial Parameters with Systolic Blood Pressure and the Cardio-Ankle Vascular Index

	Outer Diameter	Inner Diameter	Wall Thickness	Wall Reflectivity
	β (95% CI)	β (95% CI)	β (95% CI)	β (95% CI)
SBP	–0.136 (–0.151 to –0.120)	–0.136 (–0.150 to –0.121)	0.000 (–0.003 to 0.003)	–0.044 (–0.071 to –0.016)
CAVI	–0.150 (–0.408 to 0.109)	–0.135 (–0.373 to 0.102)	–0.007 (–0.050 to 0.036)	0.371 (–0.081 to 0.822)

β = regression coefficient; CAVI = cardio-ankle vascular index; CI = confidence interval; SBP = systolic blood pressure.

Multivariable linear regression models were adjusted for age, sex, body mass index, axial length, intraocular pressure, alcohol consumption, history of smoking, and histories of hypertension, diabetes mellitus, dyslipidemia, and antihypertensive medication use.

The results for the DBP models (Table S1, available at www.ophthalmologyscience.org) were consistent with those for SBP: DBP was associated with smaller OD (β = –0.252, 95% CI: –0.275 to –0.230) and smaller ID (β = –0.247, 95% CI: –0.268 to –0.226), whereas CAVI bore a positive association with wall reflectivity (β = 0.331, 95% CI: –0.119 to 0.780).

### Effect Modification by Age

Formal interaction testing (Table 3) indicated that age modified the associations of the systemic vascular indices with the retinal arterial parameters.

**Table 3:** Associations of Retinal Arterial Parameters with Systolic Blood Pressure and the Cardio-Ankle Vascular Index: Age Interaction Analysis

	Outer Diameter	Inner Diameter	Wall Thickness	Wall Reflectivity
	β (95% CI)	β (95% CI)	β (95% CI)	β (95% CI)
SBP	–0.146 (–0.162 to –0.131)	–0.145 (–0.159 to –0.130)	–0.001 (–0.003 to 0.002)	–0.044 (–0.072 to –0.016)
CAVI	–0.236 (–0.511 to 0.040)	–0.232 (–0.484 to 0.021)	–0.002 (–0.048 to 0.044)	0.180 (–0.305 to 0.666)
SBP × age	0.005 (0.004 to 0.007)	0.005 (0.004 to 0.006)	0.000 (0.000 to 0.001)	0.001 (–0.001 to 0.003)
CAVI × age	0.006 (–0.011 to 0.024)	0.009 (–0.007 to 0.025)	–0.001 (–0.004 to 0.002)	0.030 (–0.001 to 0.061)

β = regression coefficient; CAVI = cardio-ankle vascular index; CAVI × age = interaction term between the CAVI and age; CI = confidence interval; SBP = systolic blood pressure; SBP × age = interaction term between SBP and age.

Multivariable linear regression models were adjusted for age, sex, body mass index, axial length, intraocular pressure, alcohol consumption, history of smoking, and histories of hypertension, diabetes mellitus, dyslipidemia, and antihypertensive medication use.

Interaction terms were defined as the cross-product of SBP (or CAVI) and age.

For SBP × age, the interaction term was positive for both OD (β = 0.005, 95% CI: 0.004 to 0.007) and ID (β = 0.005, 95% CI: 0.004 to 0.006), consistent with weaker inverse associations in older participants.

**Figure 4:**
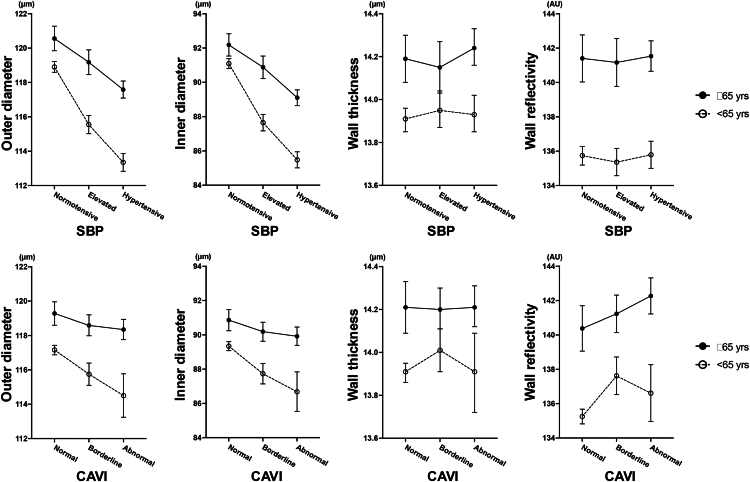
Associations of retinal arterial parameters with SBP and CAVI in age-stratified subgroups. The four OCT-derived retinal arterial parameters—OD, ID, wall thickness, and wall reflectivity—across categories of SBP and CAVI are presented, shown separately for participants aged <65 years and ≥65 years. In both age groups, higher SBP levels show associations with smaller ODs and IDs, with a more pronounced trend in the younger subgroup. In contrast, a trend toward increased wall reflectivity with higher CAVI values is visually apparent only in the older subgroup. Age cutoffs were defined based on the World Health Organization criteria and previous studies. Systolic blood pressure and CAVI were categorized as described in Figure 3. Error bars represent mean values with 95% confidence intervals. CAVI = cardio-ankle vascular index; ID = inner diameter; OD = outer diameter; SBP = systolic blood pressure.

For CAVI × age, the interaction term for wall reflectivity was positive (β = 0.030, 95% CI: –0.001 to 0.061), suggesting a stronger association in older participants. These interactions motivated subsequent analyses by predefined age groups (Table 4, Fig 4).

**Table 4:** Associations of Retinal Arterial Parameters with Systolic Blood Pressure and the Cardio-Ankle Vascular Index in Two Age Groups

	Outer Diameter	Inner Diameter	Wall Thickness	Wall Reflectivity
	β (95% CI)	β (95% CI)	β (95% CI)	β (95% CI)
Aged <65 yrs (n = 4648)				
SBP	–0.186 (–0.208 to –0.165)	–0.178 (–0.198 to –0.159)	–0.004 (–0.008 to –0.001)	–0.052 (–0.088 to –0.016)
CAVI	–0.337 (–0.730 to 0.055)	–0.365 (–0.727 to –0.002)	0.014 (–0.052 to 0.079)	–0.165 (–0.825 to 0.494)
Aged ≥65 yrs (n = 2321)				
SBP	–0.076 (–0.099 to –0.052)	–0.085 (–0.106 to –0.063)	0.005 (0.001 to 0.008)	–0.023 (–0.066 to 0.020)
CAVI	–0.094 (–0.440 to 0.253)	–0.043 (–0.359 to 0.273)	–0.025 (–0.084 to 0.033)	0.725 (0.081 to 1.370)

β = regression coefficient; CAVI = cardio-ankle vascular index; CI = confidence interval; SBP = systolic blood pressure.

Multivariable linear regression models were adjusted for age, sex, body mass index, axial length, intraocular pressure, alcohol consumption, history of smoking, and histories of hypertension, diabetes mellitus, dyslipidemia, and antihypertensive medication use.

### Analyses by Age Group

In models run separately for participants aged <65 and ≥65 years, SBP was associated with smaller OD and ID in both groups, with larger effect sizes in those aged <65 years (OD: β = –0.186, 95% CI: –0.208 to –0.165; ID: β = –0.178, 95% CI: –0.198 to –0.159) than in those aged ≥65 years (OD: β = –0.076, 95% CI: –0.099 to –0.052; ID: β = –0.085, 95% CI: –0.106 to –0.063).

In participants aged ≥65 years, CAVI was positively associated with wall reflectivity (β = 0.725, 95% CI: 0.081 to 1.370), whereas in those aged <65 years, the association was small and imprecise (β = –0.165, 95% CI: –0.825 to 0.494).

## Discussion

We found that SBP and arterial stiffness (measured via the CAVI) were differentially associated with OCT-based retinal arterial parameters, exhibiting distinct age-related patterns.

In the multivariable models created using age as a continuous variable, we observed SBP × age interactions for both arterial OD and ID as well as wall thickness. These results indicate that the inverse association between SBP and retinal arterial diameter was more pronounced in younger individuals, with the slope of the association becoming less steep with advancing age—suggesting attenuation of vasoreactive responses in older adults. Furthermore, the CAVI × age interaction for wall reflectivity indicated that the positive association between arterial stiffness and wall reflectivity strengthened with increasing age. The results of these formal interaction tests corroborated those of the age-based analyses: in participants aged <65 years, SBP showed a strong inverse association with arterial diameters, whereas in those aged ≥65 years, CAVI exhibited a marked positive association with wall reflectivity. Such complementary patterns suggest that functional vascular narrowing predominates earlier in life, whereas structural wall changes emerge as a hallmark of advanced vascular aging.^14^

The inverse association between SBP and retinal arterial diameters likely reflects dynamic vasoreactivity in response to the hemodynamic load. The stronger association observed in younger individuals could indicate preserved vascular elasticity and autoregulatory capacity, allowing dynamic changes in vessel caliber in response to pressure fluctuations. In older adults, arterial stiffening may blunt these responses, consistent with previous studies showing attenuation of microvascular reactivity with age.^30^^,^^31^ Notably, DBP also showed an inverse association with vessel caliber, supporting the notion that both SBP and DBP components influence microvascular tone.^32^

The findings pertaining to the inverse association between blood pressure and retinal arterial diameter are consistent with those of previous population-based studies using fundus photography, which reported narrower central retinal artery equivalents (CRAEs) in individuals with elevated SBP and DBP. Notably, research such as the Rotterdam Study^30^ and Funagata Study^10^ suggested that CRAE narrowing serves as both a marker and potential predictor of systemic hypertension. More recent evidence suggests that ambulatory blood pressure monitoring is an even stronger predictor of future CRAE narrowing than conventional clinic-based blood pressure measurements.^33^ Furthermore, age-related differences in this association have been observed, with the strongest effects reported in individuals aged <60 years.^34^ Our study aligns with these previous studies, showing a more pronounced association between SBP and retinal arterial diameters in participants aged <65 years.

Importantly, we expanded the existing knowledge by employing OCT-based measurements that facilitate direct and separate quantification of OD and ID and precise estimation of wall thickness. Unlike CRAE, which is derived from two-dimensional projections and offers limited structural information, OCT enables more accurate geometric evaluation of the retinal arteries. In addition to geometric measurements, OCT uniquely captures signal reflectivity within the vessel wall, which may serve as a surrogate marker of microstructural integrity, such as collagen deposition or sclerosis.^14^^,^^35^^,^^36^ This parameter is not available on fundus photography and represents a novel biomarker of early vascular remodeling. By enabling differentiation between functional vasoconstriction and structural changes, OCT-based reflectivity assessment provides additional insights into the early manifestations of microvascular damage, particularly in the context of age-related vascular stress.

In contrast to SBP, the CAVI was not associated with arterial diameters but showed a positive association with wall reflectivity in older participants. This suggests that wall reflectivity may indicate microstructural changes in the arterial wall—such as fibrosis or sclerosis—that accumulate over time and may not be apparent as changes in lumen size.^37^^,^^38^ The absence of this association in younger participants further supports the notion that structural remodeling is a feature of more advanced vascular aging. These results suggest the potential of OCT-based reflectivity as a complementary, noninvasive biomarker of early microvascular damage, which is not captured by conventional geometric parameters.

The age-related patterns observed in this study align with the current models of vascular aging. Functional changes, such as increased vascular tone and endothelial dysfunction, tend to occur earlier in life, whereas structural alterations, including medial thickening and extracellular matrix deposition, develop gradually and are largely irreversible.^39^^,^^40^ This conceptual framework supports our findings that retinal arterial narrowing was more evident in younger individuals with elevated SBP, whereas reflectivity-based changes emerged later in life, in association with arterial stiffness. Although such vascular remodeling has been well documented in large arteries, our study suggests that similar processes affect the retinal microvasculature and can be captured noninvasively by OCT.

The strengths of this study include its large sample size, use of high-resolution OCT to quantify both geometric and intensity-based vascular parameters, and age-stratified analyses grounded in prespecified hypotheses on vascular aging. Rigorous adjustment for ocular and systemic covariates further enhanced the internal validity of our findings.

### Limitations

This study has several limitations. First, given the observational and cross-sectional design, all observed relationships are associative, rather than causal, and should not be interpreted as evidence of cause and effect. Although we adjusted for key demographic and ocular variables in the multivariable analyses, the temporal direction and underlying mechanisms of these associations remain unknown. Future longitudinal studies incorporating repeated OCT-based vascular assessments, as well as experimental and histological investigations, are essential to establish temporality and strengthen causal inference. Second, blood pressure was measured at a single time point in a seated, resting state. Although this approach aligns with standard clinical practice and we used the mean of two measurements to improve reliability, it may not fully capture short-term variability because of factors such as the white-coat effect or diurnal fluctuations. Future studies incorporating repeated or ambulatory blood pressure monitoring could provide more comprehensive insights into individual hemodynamic profiles. Third, given the large sample size and multiplicity of analyses conducted, even small effect estimates with narrow CIs should be interpreted with caution. Our analyses were hypothesis-driven and restricted to predefined systemic and ocular parameters; nonetheless, no formal multiplicity adjustment was applied, and the results should be regarded as exploratory, requiring confirmation in independent cohorts.

## Conclusion

In this exploratory, cross-sectional study, OCT-based measurements of retinal arteries suggested age-related patterns in their associations with systemic vascular factors. Smaller retinal arterial diameters were associated with SBP, with stronger associations in younger participants, whereas greater wall reflectivity was associated with arterial stiffness in older participants. These findings, interpreted from effect sizes and CIs, should be considered hypothesis-generating. The ability of OCT to capture both geometric and reflectivity-based changes offers a potential means to noninvasively characterize different stages of microvascular remodeling. If confirmed in longitudinal and mechanistic studies, these OCT-derived vascular metrics may complement existing systemic assessments and contribute to earlier detection of vascular aging.

## Data Availability

The datasets generated and/or analyzed in the current study are available from the corresponding author upon reasonable request.
