# Supplementary material for: Age-Related Associations of Blood Pressure and Arterial Stiffness with Retinal Arterial Structure
Source: Ophthalmol Sci. 2025 Oct 6;6(1):100957. doi: 10.1016/j.xops.2025.100957 (PMC12651487; doi:10.1016/j.xops.2025.100957)
Supplement: Table S1 [file mmc1.pdf]

Supplementary Table 1. Associations of Retinal Arterial Parameters with Diastolic Blood Pressure and the Cardio-Ankle Vascular Index

	Outer diameter	Inner diameter	Wall thickness	Wall reflectivity
	β (95% CI)	β (95% CI)	β (95% CI)	β (95% CI)
DBP	-0.252 (-0.275 to -0.230)	-0.247 (-0.268 to -0.226)	-0.003 (-0.006 to 0.001)	-0.056 (-0.096 to -0.015)
CAVI	-0.100 (-0.353 to 0.154)	-0.096 (-0.329 to 0.136)	-0.002 (-0.044 to 0.041)	0.331 (-0.119 to 0.780)

Abbreviations: β, regression coefficient; CI, confidence interval; DBP, diastolic blood pressure; CAVI, cardio-ankle vascular index

Multivariable linear regression models were adjusted for age, sex, body mass index, axial length, intraocular pressure, alcohol consumption, history of smoking, and histories of hypertension, diabetes mellitus, dyslipidemia, and antihypertensive medication use.
